# Identification of a di-glucose conjugate of 4-hydroxybenzoic acid in bamboo cells expressing bacterial 4-hydroxycinnamoyl-CoA hydratase/lyase

**DOI:** 10.5511/plantbiotechnology.23.1218a

**Published:** 2024-03-25

**Authors:** Naoki Ube, Yasuo Kato, Taiji Nomura

**Affiliations:** 1 Biotechnology Research Center and Department of Biotechnology, Toyama Prefectural University, 5180 Kurokawa, Imizu, Toyama 939–0398, Japan

**Keywords:** bamboo cells, 4-hydroxybenzoic acid, 4-hydroxycinnamoyl-CoA hydratase/lyase, *Phyllostachys nigra*

## Abstract

Rational metabolic-flow switching is an effective strategy that we proposed for producing exogenous high-value natural products using transformed plant cells. In an earlier proof-of-concept study, we generated bamboo (*Phyllostachys nigra*; Pn) cells expressing the 4-hydroxycinnamoyl-CoA hydratase/lyase gene of *Pseudomonas putida* KT2440 (*PpHCHL*). The encoded enzyme catalyzes the formation of 4-hydroxybenzaldehyde and vanillin from *p*-coumaroyl-CoA and feruloyl-CoA, respectively. The PpHCHL-transformed Pn cells accumulated mono-glucose conjugates (glucoside and glucose ester) of 4-hydroxybenzoic acid and vanillic acid, indicating that the products (aldehydes) of the PpHCHL-catalyzed reaction were oxidized by endogenous enzyme(s) in Pn cells. In this study, we re-examined the extracts of PpHCHL-transformed Pn cells to screen for additional 4-hydroxybenzoic acid derivatives. An unidentified compound was detected exclusively in the PpHCHL-transformed Pn cells. This compound was purified via column chromatography and then identified as a di-glucose conjugate of 4-hydroxybenzoic acid (i.e., β-D-glucopyranosyl 4-*O*-β-D-glucopyranosylbenzoate), implying that some of the mono-glucose conjugates of 4-hydroxybenzoic acid were converted to the di-glucose conjugate by endogenous enzyme(s) in Pn cells. The maximum production titer of this di-glucose conjugate in the suspension-cultured cells was 0.38 g l^−1^, which was the second highest titer among the four glucose conjugates produced by the PpHCHL-transformed Pn cells. The study findings further support the utility of PpHCHL-transformed Pn cells for the bioproduction of 4-hydroxybenzoic acid and its derivatives.

Plants are a source of diverse secondary metabolites potentially useful as raw materials for the production of pharmaceuticals, flavoring agents, and fragrances. Low productivity and a long growth period often limit the commercial production of secondary metabolites using harvested plants. To overcome these limitations, several secondary metabolites have been commercially produced using plant cell culture systems (Wilson and Roberts 2012).

We previously developed a “rational metabolic-flow switching” strategy for producing exogenous secondary metabolites using plant cells (Nomura et al. 2018). As the first step of this strategy, highly active metabolic pathway(s) in the plant cells of interest is predicted by identifying the major metabolite(s) in the cells. Next, cells are transformed to introduce the exogenous gene(s) encoding the enzyme(s) required to switch the inherent active metabolic flow to the biosynthesis of the exogenous target compound(s). Bamboo (*Phyllostachys nigra*; hereafter Pn) cells were used to demonstrate the effectiveness of this strategy because they proliferate efficiently in liquid medium and accumulate substantial amounts of phenylpropanoid-derived metabolites, such as lignin and hydroxycinnamoylputrescines, depending on the culture conditions (Nomura et al. 2013; Ogita 2005; Ogita et al. 2012). We previously transformed Pn cells to stably express the barley (*Hordeum vulgare*) agmatine coumaroyltransferase gene (*HvACT*) (Nomura et al. 2018), the *Bacillus amyloliquefaciens* phenolic acid decarboxylase gene (*BaPAD*) (Kitaoka et al. 2021), and the *Pseudomonas putida* KT2440 4-hydroxycinnamoyl-CoA hydratase/lyase gene (*PpHCHL*) (Kitaoka et al. 2020). The HvACT-transformed Pn cells efficiently produced *p*-coumaroylagmatine and feruloylagmatine, which are the products of the HvACT-catalyzed reaction. In the BaPAD-transformed Pn cells, 4-vinylphenol and 4-vinylguaiacol, which are the products of the BaPAD-catalyzed reaction, accumulated as primeverose conjugates. The PpHCHL-transformed Pn cells produced 4-hydroxybenzoic acid glucose ester (4HBAGE) as the major product, but vanillic acid glucose ester (VAGE) and 4-hydroxybenzoic acid glucoside (4HBAG) were also produced, albeit at lower levels. According to these results, 4-hydroxybenzaldehyde and vanillin, which are the products of the PpHCHL-catalyzed reaction, were oxidized to 4-hydroxybenzoic acid (4HBA) and vanillic acid (VA), respectively, after which glucosyltransferase(s) present in Pn cells catalyzed the conjugation of glucose to their carboxy or hydroxy group. The PpHCHL-transformed Pn cells produced substantial amounts of 4HBAGE, VAGE, and 4HBAG, with maximum titers of 1.7, 0.17, and 0.14 g l^−1^, respectively. These three proof-of-concept studies demonstrated the efficacy of rational metabolic-flow switching for the production of exogenous metabolites as well as the suitability of Pn cells for producing phenylpropanoid-derived metabolites.

Although glycosylation hampers the accumulation of aglycon-type products in transformants, the conjugation with sugars rather enables high levels of accumulation, because sugar conjugates are usually less toxic than the corresponding aglycons and are sequestered in vacuoles. Indeed, 4HBA aglycon was undetectable in the PpHCHL-transformed Pn cells. The fact that 4HBAGE and 4HBAG accumulated in the PpHCHL-transformed Pn cells indicates that in Pn cells, glucose may be conjugated to the carboxy and hydroxy groups of 4HBA. However, we only identified mono-glucose conjugates, suggesting we may have overlooked the accumulation of the di-glucose conjugate of 4HBA. To assess this possibility, in the present study, we re-analyzed the extracts of PpHCHL-transformed Pn cells, which revealed the substantial accumulation of the di-glucose conjugate of 4HBA. The study results may be relevant to the bioproduction of 4HBA, an industrially useful intermediate compound.

In this study, Pn suspension cells, which are currently available from the RIKEN BioResource Research Center (no. rpc00047; https://web.brc.riken.jp/ (Accessed Sep. 10, 2023)), were maintained in modified Murashige and Skoog (MS) liquid medium supplemented with 680 mg l^−1^ KH_2_PO_4_, 10 µM 4-amino-3,5,6-trichloropyridine-2-carboxylic acid (picloram), and 3% (w/v) sucrose (Murashige and Skoog 1962; Ogita 2005; Ogita et al. 2011). This medium strongly promotes the proliferation of Pn cells and is referred to as PR medium. The cells were subcultured in 100 ml liquid medium in a 300 ml Erlenmeyer flask and maintained on a rotary shaker (100 rpm) in darkness at 25°C. The cells were subcultured every 2 weeks by adjusting the initial sedimented cell volume (SCV) to 5% as previously described (Ogita et al. 2011)

PpHCHL (GenBank accession no. SKC00150)-transformed Pn cell lines 20 and 28 as well as PpHCHL-negative lines 23 and 26, which we generated previously (Kitaoka et al. 2020), were maintained as calli on PR medium supplemented with 100 mg l^−1^ hygromycin B and solidified with 0.3% (w/v) gellan gum and as suspension cells in liquid PR medium without hygromycin B. The subcultured suspension cells of the wild type (WT) and transformants were transferred to the following fresh media, with an initial SCV of 5%: PR medium, half-strength MS medium containing 3% (w/v) sucrose (lignification-1; LG1 medium), and half-strength MS medium containing 3% (w/v) sucrose and 10 µM 6-benzyladenine (lignification-2; LG2 medium) (Nomura et al. 2018, 2013). The cells were cultured as described above, with samples collected every 5 days for 30 days as previously described (Nomura et al. 2013).

To analyze the Pn cell metabolites, the suspension cell extracts of the PpHCHL-transformed lines and the PpHCHL-negative lines as well as the WT cultured under PR, LG1, and LG2 conditions were prepared by sonication (10 min) using 10 volumes of 80% (v/v) MeOH/2% (v/v) AcOH. After the extracts were centrifuged (21,500×g, 10 min, 4°C), the supernatants were analyzed using an HPLC system (column, Mightysil RP-18 GP Aqua, 5 µm, 4.6×250 mm, Kanto Chemical Co. Inc., Tokyo, Japan; solvent, 5% (v/v) MeOH/0.1% trifluoroacetic acid; flow rate, 0.8 ml min^−1^; detection wavelength, 280 nm; column temperature, 40°C).

To isolate compound **1**, PpHCHL-transformed cell line 20 was cultured for 2 weeks under PR conditions. The cells were collected from 1 l liquid culture (10 flasks each containing 100 ml culture) on filter paper via vacuum filtration. The collected cells (194 g fresh weight, FW) were mixed with 2 l of 80% (v/v) MeOH and sonicated for 60 min at room temperature. The resulting extract was filtered through a diatomite pad (Radiolite #3000; Showa Chemical Industry, Tokyo, Japan) and then the filtrate was concentrated to approximately 200 ml and washed three times with *n*-hexane. The aqueous layer was concentrated and applied to an octadecylsilyl (ODS) column (Cosmosil 75C_18_-OPN, Nacalai Tesque, Kyoto, Japan; 5×8 cm; 150 ml) equilibrated with water. For the sequential elution of compounds, 0%, 5%, 10%, and 20% (v/v) MeOH (450 ml per solvent) were added to the column. The flow-through and 0% MeOH fraction were combined, concentrated to approximately 20 ml, and applied to a charcoal column (charcoal activated for chromatography, 20–150 mesh, Nacalai Tesque; 4×16 cm; 200 ml) equilibrated with 50% (v/v) MeOH. For the sequential elution of compounds, 50%, 60%, 70%, 80%, and 100% (v/v) MeOH (600 ml per solvent) were added to the column. The 60%, 70%, and 80% MeOH fractions were combined, concentrated to approximately 5 ml, passed through a membrane filter (Millex-HV, 0.45 µm; Merck, Darmstadt, Germany), and subjected to reversed-phase preparative HPLC (column, TSKgel ODS-80Ts, 5 µm, 20×250 mm, Tosoh, Tokyo, Japan; solvent, 10% (v/v) MeOH; flow rate, 5 ml min^−1^; detection wavelength, 280 nm). Compound **1** was eluted at 35 min; the collected fraction was concentrated and lyophilized to obtain 80.1 mg of **1** as a colorless powder. The NMR spectra of **1** were recorded in CD_3_OD using the AVANCE 400 spectrometer (Bruker, Billerica, MA, USA). The high-resolution electrospray ionization time-of-flight mass spectrometry (HR-ESI-TOF-MS) analysis was performed using the micrOTOF focus spectrometer (Bruker). The UV spectrum and specific optical rotation were measured using the UV-1800 spectrophotometer (Shimadzu, Kyoto, Japan) and the P-1030 Polarimeter (Jasco, Tokyo, Japan), respectively.

The spectral properties of β-D-glucopyranosyl 4-*O*-β-D-glucopyranosylbenzoate (4HBAGGE, **1**) were as follows: HR-ESI-TOF-MS (Supplementary Figure S1) (positive) *m*/*z* 485.1267 [M+Na]^+^ (calcd. for C_19_H_26_O_13_Na^+^, 485.1266); UV (MeOH) λ_max_ (log ε) 203 nm (4.21), 253 nm (4.27); [α]_D_^22^=−50.7° (*c* 0.30, MeOH); ^1^H-NMR (400 MHz, CD_3_OD, Supplementary Figure S2A) δ (ppm) 3.40–3.43 (m, 4H, H-3′, 4′, 3″, 4 ″), 3.45–3.51 (m, 4H, H-2′, 5′, 2″, 5″), 3.70 (m, 2H, H-6a′, 6a″), 3.87 (m, 2H, H-6b′, 6b″), 5.03 (d, 1H, *J*=7.5, H-1″), 5.70 (d, 1H, *J*=8.0, H-1′), 7.17 (d, 2H, *J*=8.9, H-3, 5), 8.05 (d, 2H, *J*=8.9, H-2, 6); ^13^C-NMR (100 MHz, CD_3_OD, Supplementary Figure S2B) δ (ppm) 60.9 (C-6′ or 6″), 61.0 (C-6′ or 6″), 69.7 (C-4′ or 4″), 69.8 (C-4′ or 4″), 72.7 (C-2′ or 2″), 73.4 (C-2′ or 2″), 76.5 (C-3′ or 3″), 76.7 (C-3′ or 3″), 76.9 (C-5′ or 5″), 77.5 (C-5′ or 5″), 94.8 (C-1′), 100.2 (C-1″), 115.9 (C-3, 5), 123.0 (C-1), 131.6 (C-2, 6), 161.9 (C-4), 165.0 (C-7). The HMBC, HSQC, and COSY spectra are presented in Supplementary Figure S2C, D, and E, respectively.

We previously detected the accumulation of the mono-glucose conjugates of 4HBA and VA (e.g., 4HBAG, 4HBAGE, and VAGE) in two PpHCHL-transformed lines (20 and 28), but not in two PpHCHL-negative lines (23 and 28) or in the WT. When the methanolic extracts of the suspension cells were analyzed by HPLC under conditions different from those in our previous study (Kitaoka et al. 2020), compound **1** was eluted at 8.3 min for PpHCHL-transformed line 20, but not for PpHCHL-negative line 23 or the WT (Figure 1). Moreover, the peak retention time of **1** was earlier than those of 4HBAG and 4HBAGE, indicating **1** is more hydrophilic than the other two compounds. Therefore, **1** was considered to be a possible di-glucose conjugate of 4HBA, which we overlooked previously.

**Figure 1:**
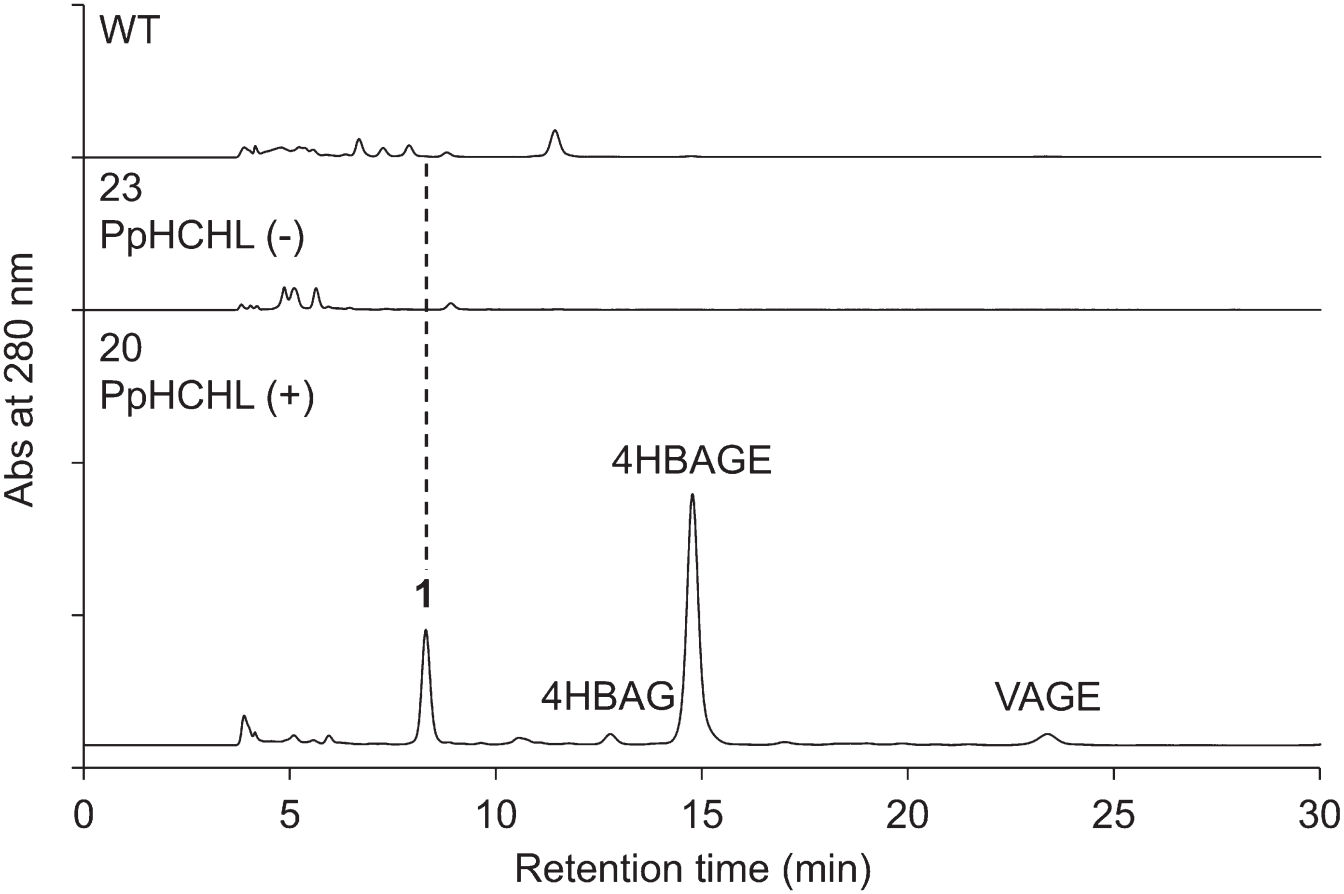
Figure 1. Analysis of the metabolites in the extracts of PpHCHL-transformed Pn cells. HPLC chromatograms are presented for the extracts of the wild-type (WT), PpHCHL-negative line 23, and PpHCHL-transformed line 20 cells cultured for 2 weeks under proliferation (PR) conditions.

We purified 80.1 mg of **1** from 194 g of the suspension cells of PpHCHL-transformed line 20 cultured for 2 weeks under PR conditions. The molecular formula of **1** (C_19_H_26_O_13_) was determined on the basis of the HR-ESI-TOF-MS data (*m*/*z* 485.1267 [M+Na]^+^, Supplementary Figure S1). The ^1^H- and ^13^C-NMR spectra of **1** (Supplementary Figure S2A, B) were similar to those of 4HBAGE and 4HBAG (Kitaoka et al. 2020). However, the ^1^H-NMR spectrum of **1** differed from those of 4HBAG and 4HBAGE in the following two ways: (1) presence of two anomeric proton signals at δ_H_ 5.03 ppm and 5.70 ppm, which was in contrast to the presence of one anomeric proton signal for 4HBAG and 4HBAGE; (2) 2-fold increase in the integral values of the proton signals for the glucose moiety. These differences and the predicted molecular formula suggested that **1** had two glucose moieties conjugated to 4HBA. The analysis of the sugar composition of **1** verified that the sugar moiety of **1** is composed of only glucose (Supplementary Figure S3). In the HMBC spectrum of **1** (Figure 2, Supplementary Figure S2C), two anomeric protons at δ_H_ 5.03 ppm for H-1″ and 5.70 ppm for H-1′ showed cross-peaks with carbon signals at δ_C_ 161.9 ppm for C-4 and 165.0 ppm for C-7, respectively, indicating that each of the two glucose moieties was connected to 4HBA, with linkages of 4-*O*-1″ (glucoside) and 7-*O*-1′ (glucose ester). The β configurations of the anomeric carbons (C-1′ and C-1″) of the two glucose moieties were determined on the basis of the coupling constants of the doublet anomeric protons (*J*_H-1′_=7.5 Hz, *J*_H-1″_=8.0 Hz). In addition, the specific optical rotation value of **1** ([α]_D_^22^ −50.7°) was comparable to a previously reported value ([α]_D_^25^ −18.4°) (Braham et al. 2005). Considered together, we concluded that compound **1** is β-D-glucopyranosyl 4-*O*-β-D-glucopyranosylbenzoate (4-hydroxybenzoic acid glucoside glucose ester, 4HBAGGE, Figure 2). The proton and carbon signals in the ^1^H- and ^13^C-NMR spectra were assigned using the HMBC, HSQC, and COSY spectra (Supplementary Figure S2C–E). The spectral data were in accordance with published data (Braham et al. 2005).

**Figure 2:**
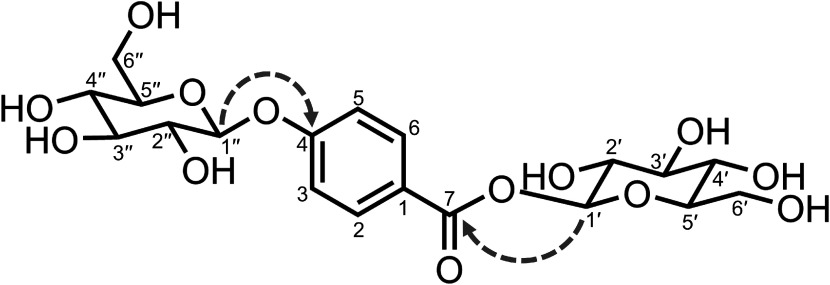
Figure 2. Chemical structure of 4-hydroxybenzoic acid glucoside glucose ester (4HBAGGE, **1**). Key HMBC correlations are indicated by dotted arrows from ^1^H to ^13^C.

The time-course changes in the 4HBAGGE contents of the suspension cells of PpHCHL-transformed lines 20 and 28 were examined under PR, LG1, and LG2 conditions (Figure 3A). The 4HBAGGE content was greater in line 20 than in line 28. The accumulation of 4HBAGGE in line 20 was enhanced under LG1 and LG2 conditions. The difference in the 4HBAGGE contents of lines 20 and 28 was consistent with the differences in the 4HBAG, 4HBAGE, and VAGE contents, which were due to the lower PpHCHL activities in line 28 than in line 20 (Kitaoka et al. 2020). The 4HBAGGE content in line 20 peaked at 24.6 µmol g^−1^ FW on day 10 under LG1 conditions. The 4HBAGGE production titer of line 20 peaked at 0.38 g l^−1^ on day 15 under LG1 conditions (Figure 3B).

**Figure 3:**
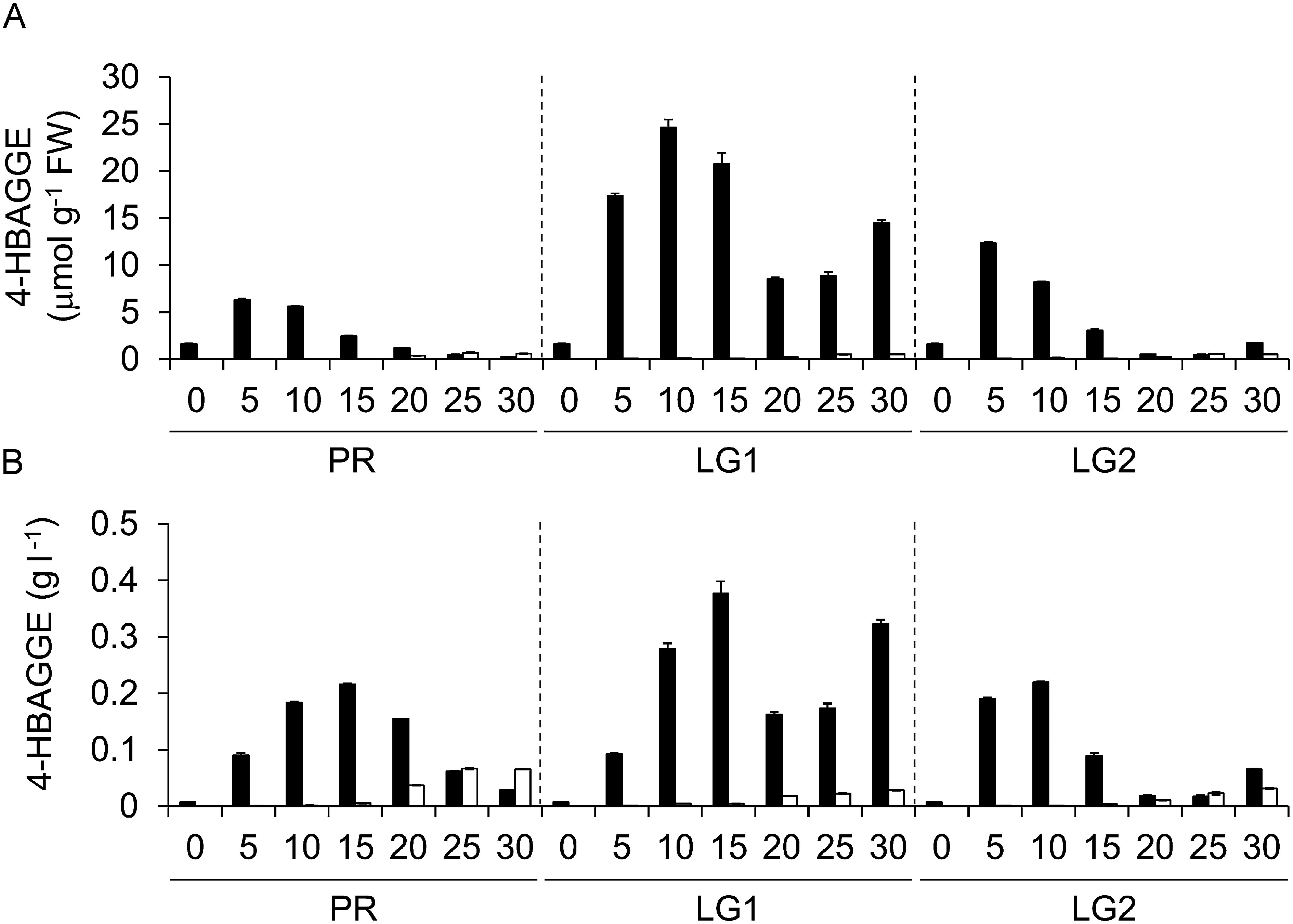
Figure 3. 4HBAGGE production in PpHCHL-transformed suspension cells. (A) Content and (B) production titer of 4HBAGGE in the suspension cells of lines 20 (black bars) and 28 (white bars) cultured under proliferation (PR), lignification-1 (LG1), and lignification-2 (LG2) conditions. The production titer was calculated by multiplying the content by the cell fresh weight (FW) for a 1 l culture. Data are presented as the mean±SD (*n*=3).

We previously reported that PpHCHL-transformed Pn cells (line 20) produce 4HBAGE, VAGE, and 4HBAG, with maximum production titers of 1.7, 0.17, and 0.14 g l^−1^, respectively. In the present study, in addition to these mono-glucose conjugates, the di-glucose conjugate of 4HBA (i.e., 4HBAGGE) was detected in the PpHCHL-transformed cells (line 20); the maximum production titer (0.38 g l^−1^) was a quarter of that of 4HBAGE, but the second highest among the four 4HBA/VA derivatives. This finding further highlights the utility of Pn cells for the production of phenylpropanoid-derived metabolites via rational metabolic-flow switching. Although glucose conjugates of 4HBA, including 4HBAGGE, 4HBAGE, and 4HBAG, have limited antioxidative activities (Braham et al. 2005), synthetic 4HBA esters (i.e., parabens) are extensively used as preservatives in the cosmetic and pharmaceutical industries. Moreover, 4HBA is a useful intermediate for the synthesis of high-value bioproducts (Wang et al. 2018). The bioproduction of glucose conjugates of 4HBA using Pn cells may facilitate the petroleum-free production of 4HBA; treatment of the extracts of PpHCHL-transformed Pn cells with a commercial β-glycosidase can easily form free 4HBA.

The accumulation of 4HBAGGE in PpHCHL-transformed line 20 was enhanced under LG1 and LG2 conditions (Figure 3A), which was consistent with the reported accumulation profiles of the other glucose ester metabolites 4HBAGE and VAGE (Kitaoka et al. 2020). In contrast, 4HBAG (i.e., glucoside), but not 4HBAGE (i.e., glucose ester), was detected at similar levels under all culture conditions (Kitaoka et al. 2020), likely because of the presence of glucose ester-forming and glucoside-forming glucosyltransferases; the expression of the former was induced under LG1 and LG2 conditions, but the expression of the latter was the same in all culture conditions. Glucose esterification of 4HBAG and glucosylation of 4HBAGE (Figure 4) are the two pathways for the formation of 4HBAGGE, but the former pathway likely takes precedence over the latter pathway.

**Figure 4:**
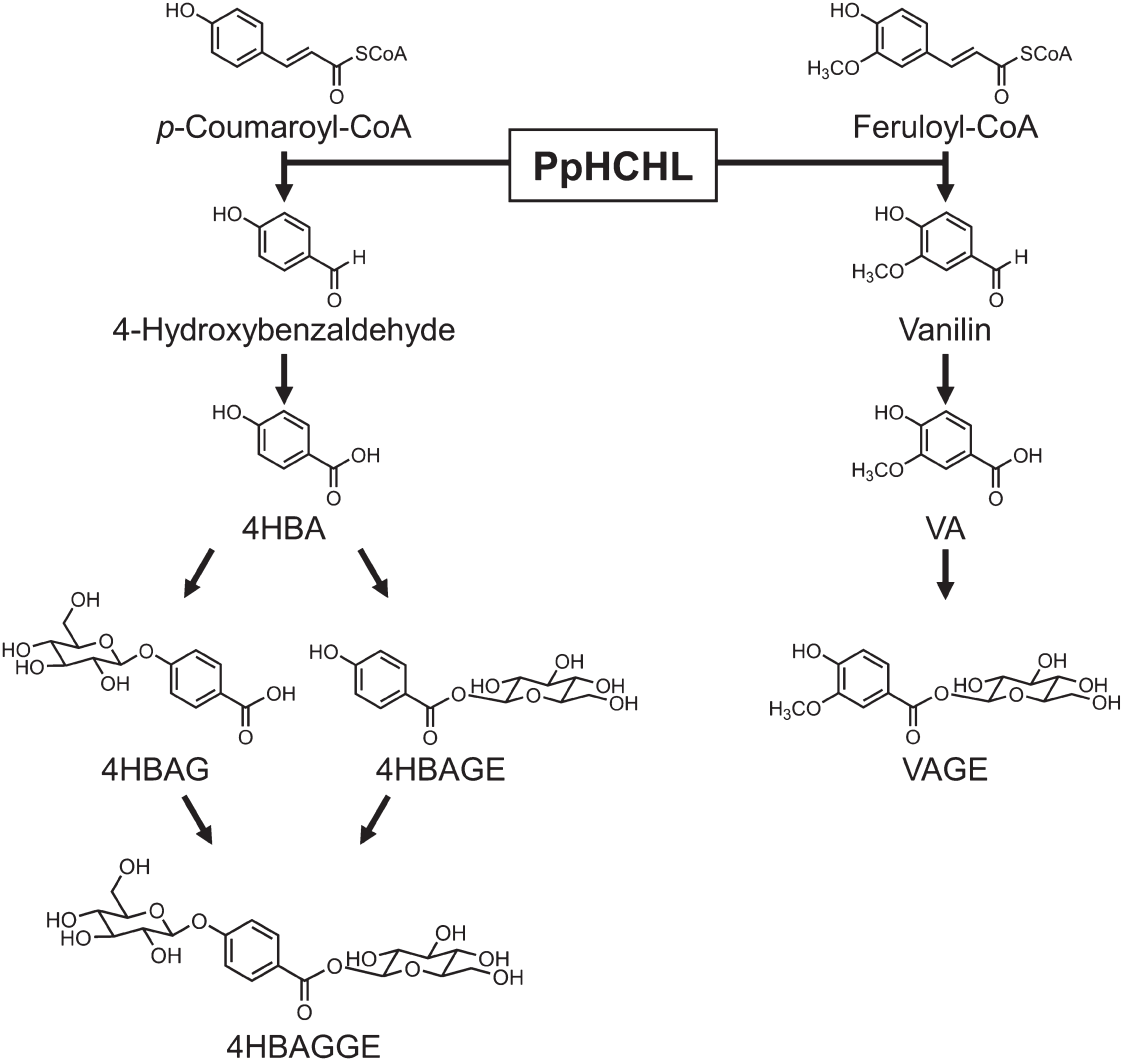
Figure 4. Putative 4HBAGGE production pathway.

In the PpHCHL-transformed Pn cells, 4HBA was modified by the addition of one or two units of glucose. Additionally, 4HBA derivatives conjugated with other sugar molecules were undetectable. However, in the BaPAD-transformed Pn cells that were generated for the bioproduction of vinylphenols, the target compounds were conjugated to primeverose, a disaccharide composed of glucose and xylose (Kitaoka et al. 2021). Moreover, in HvACT-transformed Pn cells, the products of the HvACT-catalyzed reaction, *p*-coumaroylagmatine and feruloylagmatine, accumulated in their free forms even though they contain phenolic hydroxy groups, which may be targeted for glycosylation (Nomura et al. 2018). The factors determining the glycosylation patterns of exogenously produced compounds are unknown, but the substrate specificities of the endogenous glycosyltransferases in Pn cells may be one of them. For the rational design of the bioproduction of exogenous compounds in Pn cells, glycosyltransferases will need to be identified and characterized.

## Abbreviations

BaPAD: *Bacillus amyloliquefaciens* phenolic acid decarboxylase
4HBA: 4-hydroxybenzoic acid
4HBAG: 4-hydroxybenzoic acid glucoside
4HBAGE: 4-hydroxybenzoic acid glucose ester
4HBAGGE: 4-hydroxybenzoic acid glucoside glucose ester
FW: fresh weight
HvACT: *Hordeum vulgare* agmatine coumaroyltransferase
LG1: lignification-1
LG2: lignification-2
ODS: octadecylsilyl
Pn: *Phyllostachys nigra*
PpHCHL: *Pseudomonas putida* hydroxycinnamoyl-CoA hydratase/lyase
PR: proliferation
SCV: sedimented cell volume
VA: vanillic acid
VAGE: vanillic acid glucose ester
WT: wild-type

## Description of Supplementary Files

Supplementary Figure S1. Mass spectrum (HR-ESI-TOF-MS) of 4HBAGGE (1).

Supplementary Figure S2. NMR spectra of 4HBAGGE(1).

Supplementary Figure S3. Analysis of the sugar composition of 4HBAGGE (1).
