# Supplementary material for: Identification of a di-glucose conjugate of 4-hydroxybenzoic acid in bamboo cells expressing bacterial 4-hydroxycinnamoyl-CoA hydratase/lyase
Source: Plant Biotechnol (Tokyo). 2024 Mar 25;41(1):83–7. doi: 10.5511/plantbiotechnology.23.1218a (PMC11500581; doi:10.5511/plantbiotechnology.23.1218a)
Supplement: Supplementary Data [file plantbiotechnology-41-1-23.1218a-s001.pdf]

Supplementary File

Identification of a di-glucose conjugate of 4-hydroxybenzoic acid in bamboo cells expressing bacterial 4-hydroxycinnamoyl-CoA hydratase/lyase

Naoki Ube[1,*], Yasuo Kato[1], Taiji Nomura[1]

[1]Biotechnology Research Center and Department of Biotechnology, Toyama Prefectural University, 5180 Kurokawa, Imizu, Toyama 939-0398, Japan

*Corresponding author. Tel.: +81-766-56-7500 (ext. 1517)

E-mail address: nube@pu-toyama.ac.jp

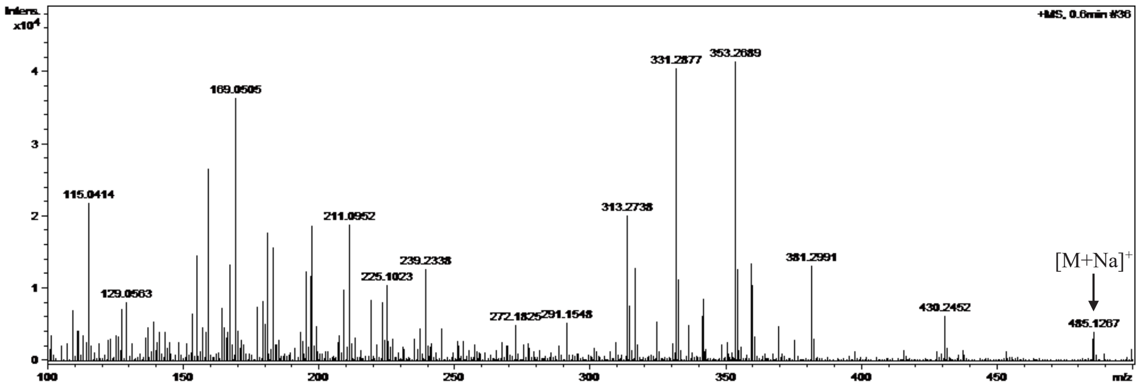

Figure S1. Mass spectrum (HR-ESI-TOF-MS) of 4HBAGGE (1).

A

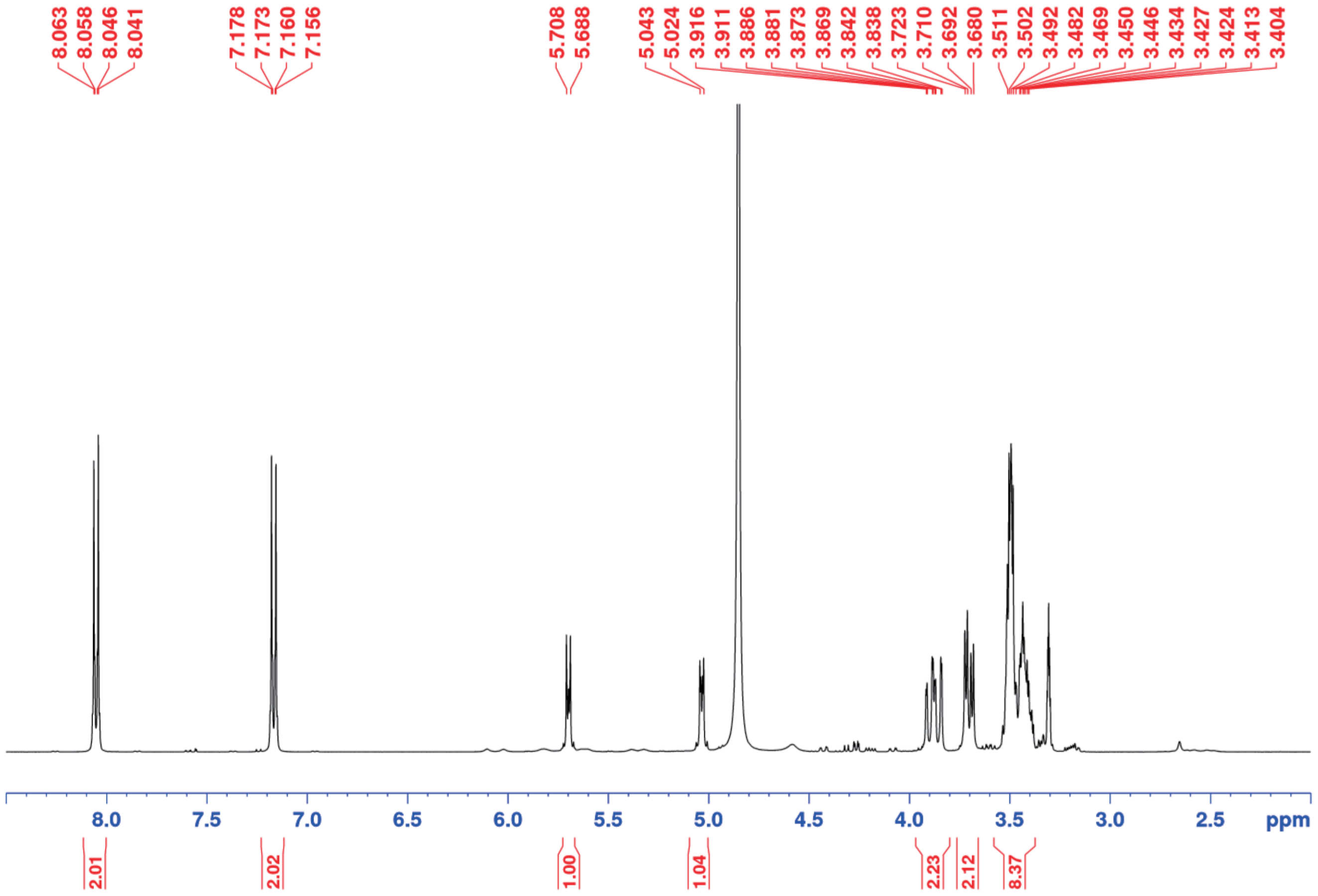

Figure S2. NMR spectra of 4HBAGGE (1).

(A) ^{1}H-NMR spectrum (400 MHz, CD_3OD)

B

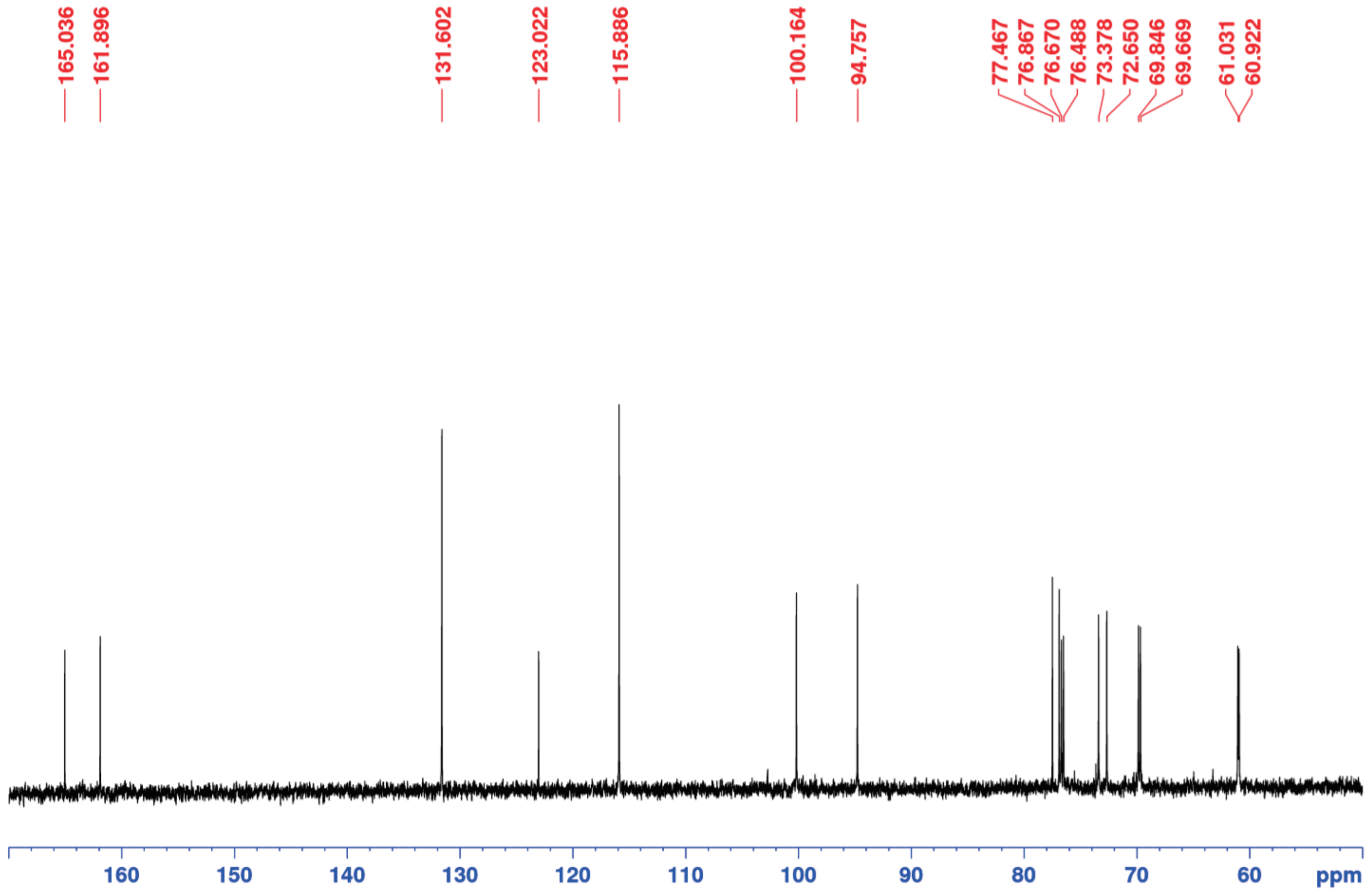

Figure S2. (continued) NMR spectra of 4HBAGGE (1).

(B) ^{13}C-NMR spectrum (100 MHz, CD_3OD)

C

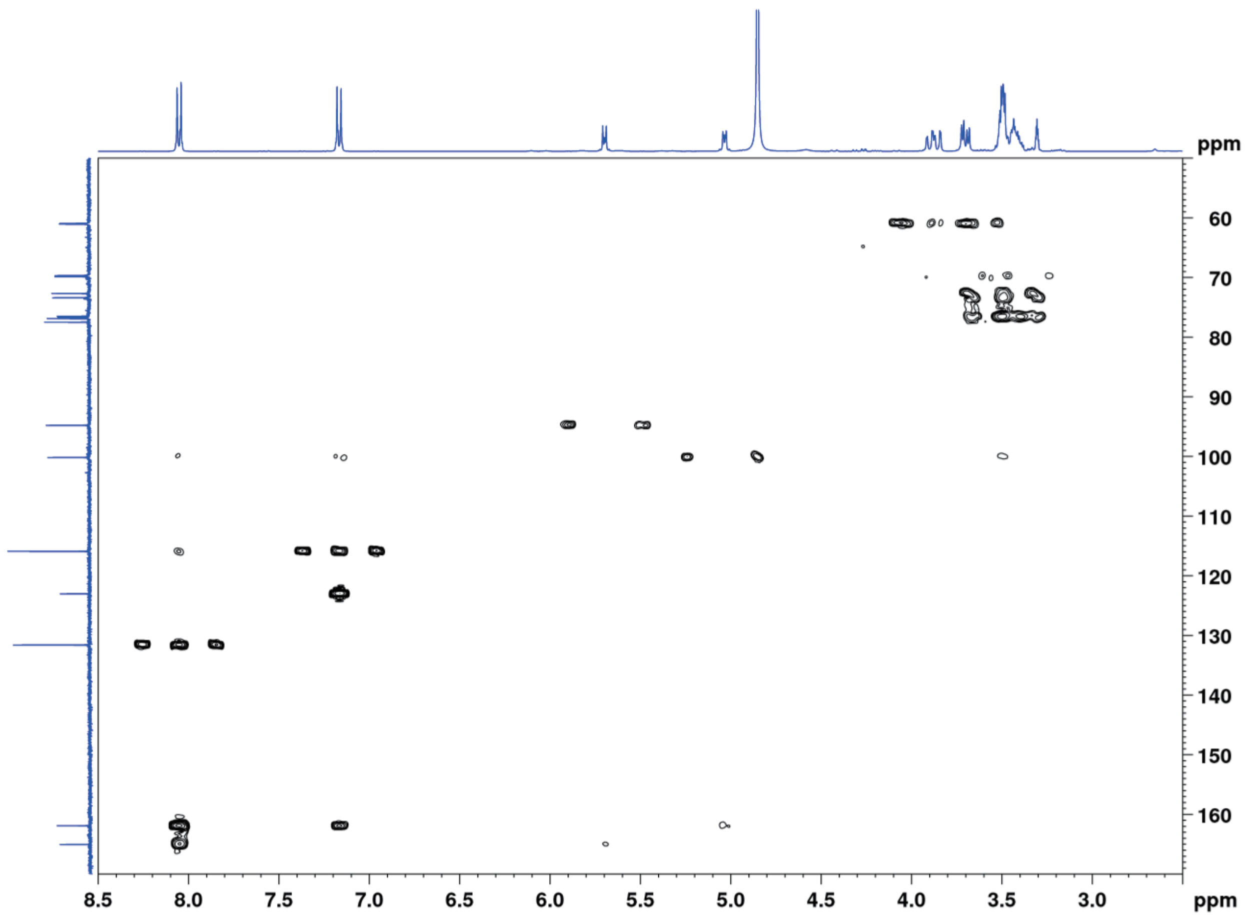

Figure S2. (continued) NMR spectra of 4HBAGGE (1).

(C) HMBC spectrum

D

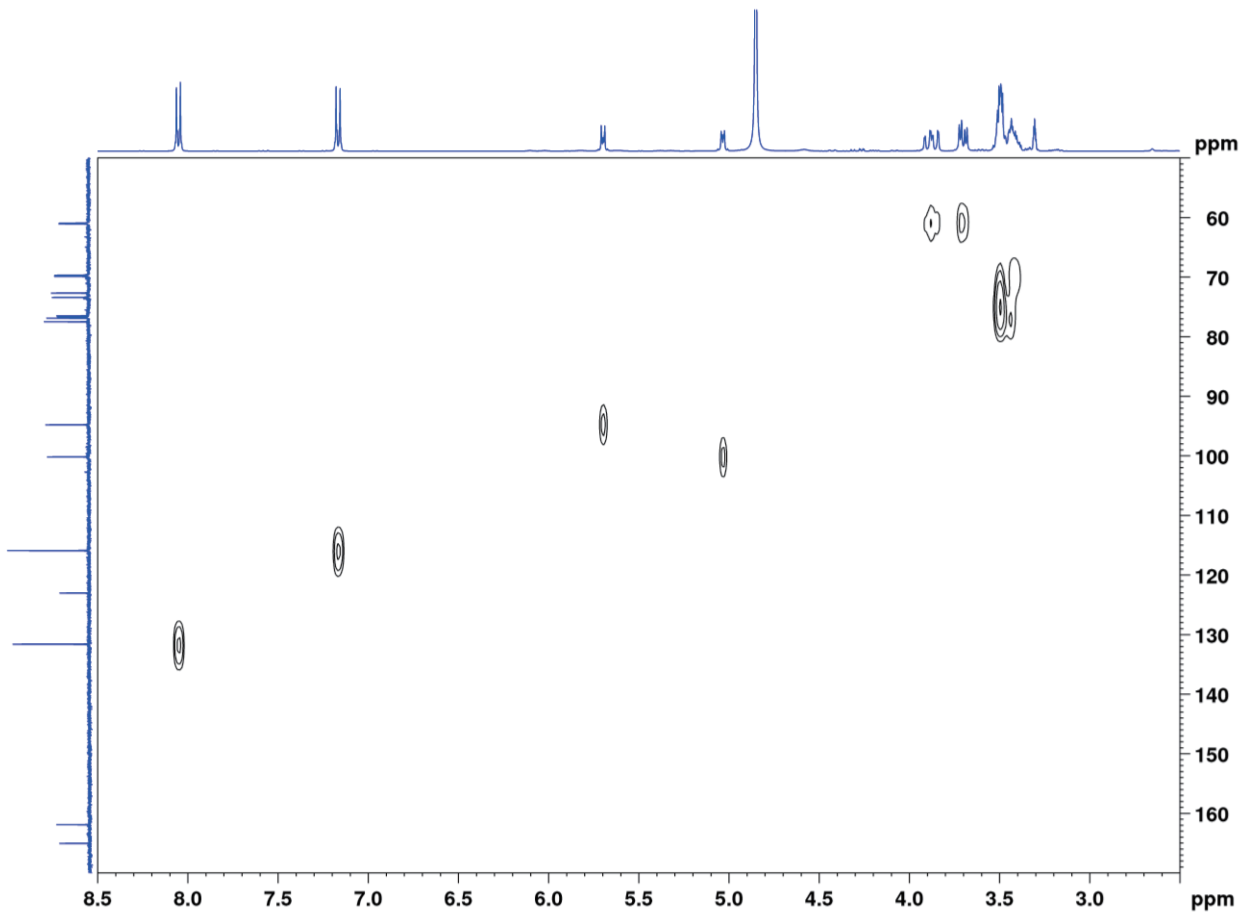

Figure S2. (continued) NMR spectra of 4HBAGGE (1).

(D) HSQC spectrum

E

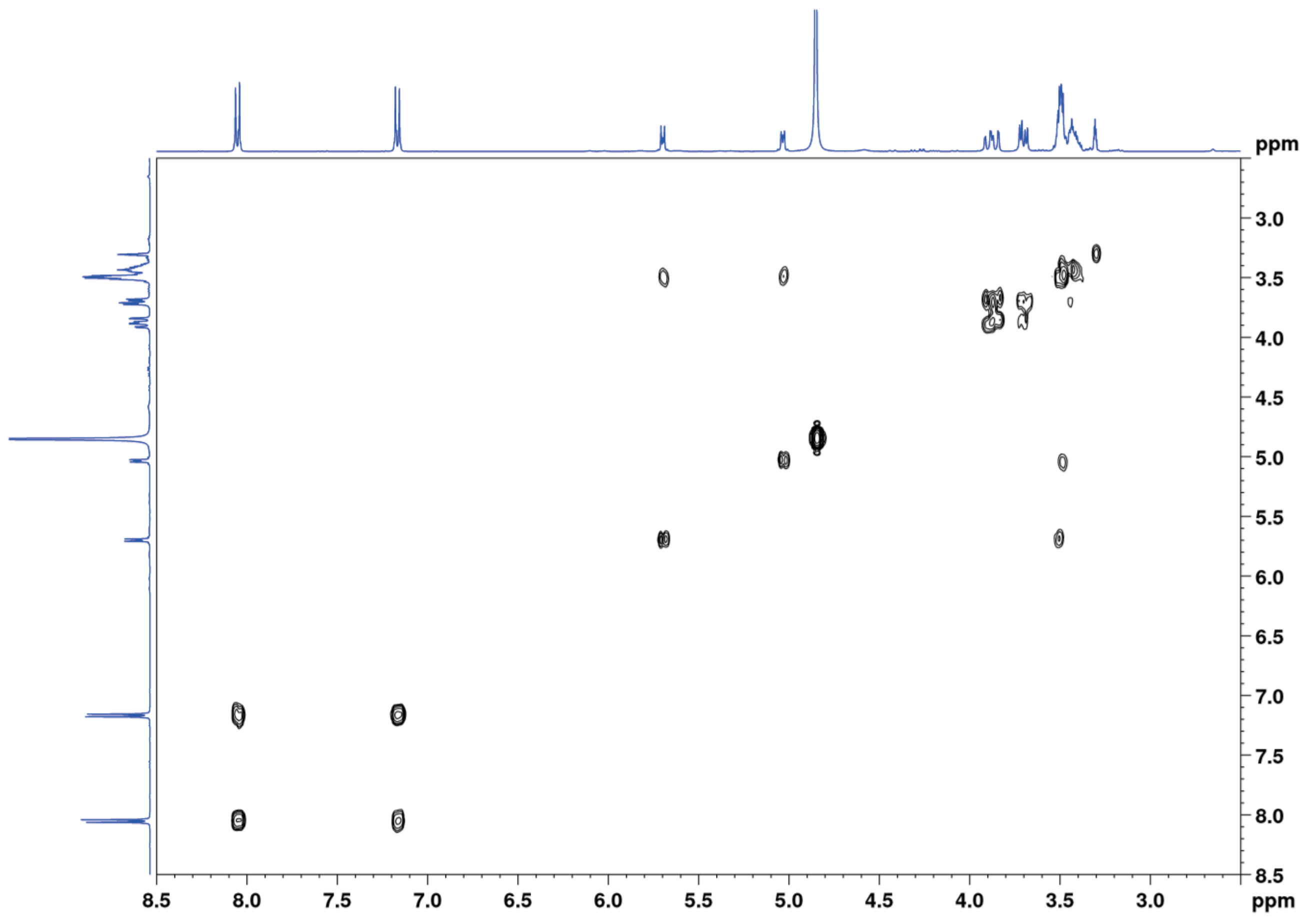

Figure S2. (continued) NMR spectra of 4HBAGGE (1).

(E) COSY spectrum

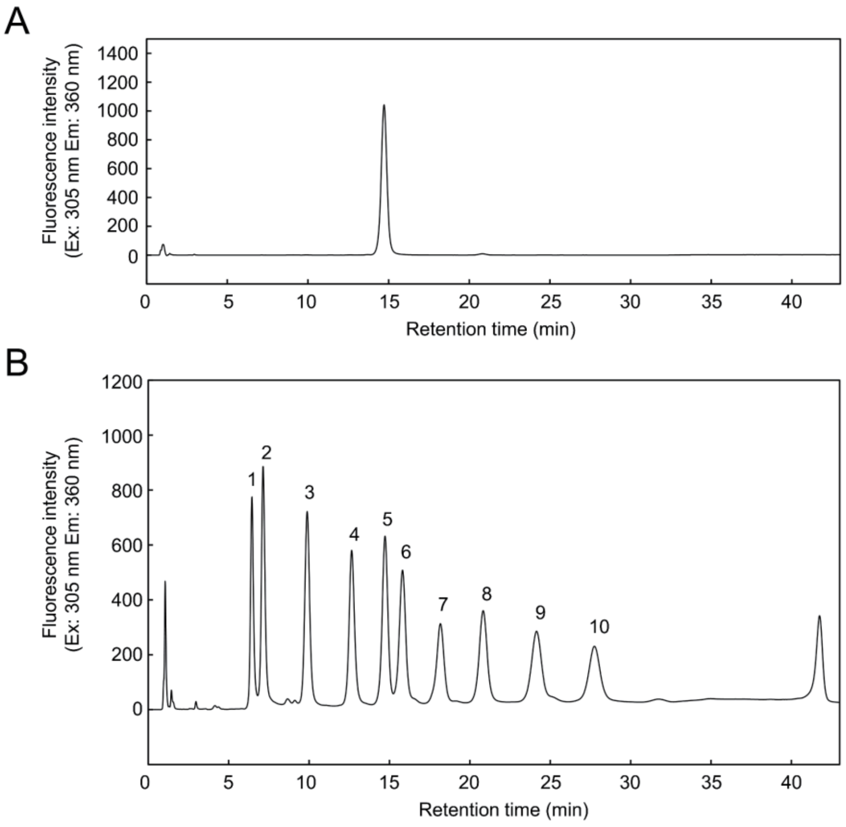

Figure S3. Analysis of the sugar composition of 4HBAGGE (1). The analysis was performed using 1.1 mg purified 4HBAGGE (**1**) at ProteNova Co., Ltd. (Kagawa, Japan) as previously described (Kitaoka et al. 2021). The hydrolyzed products of 4HBAGGE (**1**) (**A**) and the monosaccharide mixture (**B**) were labeled with the 4-aminobenzoic acid ethyl ester (ABEE) reagent. The ABEE-labeled sugars were subjected to an HPLC analysis (fluorescence detection; excitation wavelength: 305 nm, emission wavelength: 360 nm). Peaks 1–10 correspond to the following monosaccharides: 1, glucuronic acid; 2, galacturonic acid; 3, galactose; 4, mannose; 5, glucose; 6, arabinose; 7, ribose; 8, xylose; 9, fucose; 10, rhamnose.

Reference

Kitaoka N, Nomura T, Ogita S, Kato Y (2021) Bioproduction of 4-vinylphenol and 4-vinylguaiacol β-primeverosides using transformed bamboo cells expressing bacterial phenolic acid decarboxylase. *Appl Biochem Biotechnol* 193: 2061–2075
